# Investigation of the biomarkers involved in ectopic ossification: The shared mechanism in ossification of the spinal ligament

**DOI:** 10.3389/fgene.2022.991834

**Published:** 2022-10-07

**Authors:** Jiachen Liu, Yunxia Chen, Xiuqi Shan, Huan Wang

**Affiliations:** ^1^ Department of Orthopedics, Shengjing Hospital of China Medical University, Shenyang, China; ^2^ Department of Endocrinology, Cangzhou People’s Hospital, Cangzhou, China

**Keywords:** ossification of the posterior longitudinal ligament, ossification of the ligamentum flavum, M6A, immune cell infiltration, shared biomarkers

## Abstract

**Background:** Ossification of the posterior longitudinal ligament (OPLL) and ossification of the ligamentum flavum (OLF) are multifactor diseases characterized by progressively ectopic ossification in the spinal ligament. However, the shared ossification mechanism of OPLL and OLF remains to be elucidated. The study aims to investigate the common biomarkers related to ectopic ossification and the potential molecular regulatory mechanism.

**Methods:** Microarray and RNA-seq datasets were obtained from Gene Expression Omnibus (GEO) database. The differentially expressed genes (DEGs) from OPLL and OLF were identified to construct the protein-protein interaction (PPI) network. Furthermore, the hub intersection genes were screened and the diagnostic performance was assessed in the external OLF and OPLL cohorts. We also depicted the landscape of immune cell infiltration and m6A modification meanwhile further estimating the relationship with BMP4.

**Results:** A total of nine up-regulated DEGs and 11 down-regulated DEGs were identified to construct the PPI networks. The integrative bioinformatic analysis defined five hub genes (BMP4, ADAMTS4, HBEGF, IL11, and HAS2) as the common risk biomarkers. Among them, BMP4 was the core target. ROC analysis demonstrated a high diagnostic value of the hub genes. Moreover, activated B cells were recognized as shared differential immune infiltrating cells and significantly associated with BMP4 in OPLL and OLF. Meanwhile, a strong correlation was detected between the expression pattern of the m6A regulator METTL3 and BMP4.

**Conclusion:** This study first identified BMP4 as the shared core biomarker in the development of OPLL and OLF. Activated B cells and m6A writer METTL3 might be involved in the osteogenesis process mediated by BMP4. Our findings provide insights into the pathogenesis in the ossification of the spinal ligament and unveil the potential therapeutic targets.

## Introduction

Ossification of the spinal ligament (OSL) features an aggravated pathological process of ectopic ossification in ligamentous tissues. Ossification of the posterior longitudinal ligament (OPLL) and ossification of the ligamentum flavum (OLF) are common subtypes of OSL, which usually lead to spinal cord compression and even myelopathy (Inamasu et al., 2006). OPLL usually occurs in the cervical spine which is relatively common in East Asia (Aljuboori and Boakye, 2019). However, OLF most occurred in the thoracic and lumbar segments of the spine. The treatments for symptomatic OPLL/OLF are limited, and no effective treatments other than surgery are available. Cross-sectional studies based on CT scans demonstrated that patients suffering from cervical OPLL are often accompanied by thoracic OLF (Kawaguchi et al., 2016; Singh et al., 2021). Previous studies laid emphasis on the genetic background and explored the susceptibility loci in OLF and OPLL respectively (Liu et al., 2010; Nakajima et al., 2014; Fan et al., 2020). However, few studies focused on the shared phenotypes and signal pathways in the mechanism of OSL at the transcriptional level. Therefore, more perspectives are required to identify the shared biomarkers and potential pathways correlated with the progression of the ossification in spinal ligaments.

Epigenetic effects on bone homeostasis have been investigated in recent years. N6-methyladenosine methylation (m6A) is considered the most common epigenetic modification on mRNAs (Wang et al., 2015). Increasing evidence has suggested the crucial role of m6A played in the pathogenesis of OPLL and OLF (Wang et al., 2020; Yuan et al., 2021). Activation of the inflammatory response may contribute to stimulating osteoblastic differentiation of ligament fibroblasts in OPLL and OLF (Ren et al., 2013; Chen et al., 2017). Thus, investigating the epigenetic modification and immune response provides novel insight into the development of OSL.

In the present study, we first identified the shared biomarkers of OPLL and OLF through the microarray and RNA-seq datasets. The protein-protein interaction (PPI) and enrichment analysis were applied to elucidate the key biomarkers and biological function in OSL. Moreover, we assessed the diagnostic performance of hub genes and further evaluate the relationship between immune cell infiltration and m6A regulators. The results preliminarily inferred the shared biomarkers and pathways involved in the pathogenesis of OSL, providing insights into novel therapeutic targets.

## Methods

### Data collection and preprocessing

The datasets GSE153829, GSE69787, GSE106253, GSE83569, and GSE181716 were obtained in the Gene Expression Omnibus (GEO) database (http://www.ncbi.nlm.nih.gov/gds/). The RNA-seq dataset GSE69787 was processed by the transcriptome annotation file and converted to transcripts per kilobase million (TPM). The heterogeneity among different experimental batches was removed by ComBat algorithm from the “sva” package (Leek et al., 2012). Differentially expressed genes (DEGs) were screened according to the cutoff criteria |logFC (fold change)| >1 and *p* value < 0.05 with the limma package in R. The DEGs in OPLL and OLF were intersected to obtain the common DEGs in OSL. GSE181716 and GSE83569 were applied for external validation. The detail of the included datasets was listed in Supplementary Table S1.

### Construction of PPI network and hub DEGs identification

PPI network was developed through the STRING database (https://string-db.org/), which predicts the protein interactions of the shared DEGs (von Mering et al., 2003). The hub genes were determined by the MCODE network analyzer and the Cytohubba plugin the network was visualized in Cytoscape software. Principal component analysis (PCA) for the expression pattern of hub DEGs was visualized by a 3D scatterplot. The diagnostic performance of hub genes was verified in the OPLL and OLF external datasets by the receiver operating curve (ROC) curve and bootstrap resampling algorithm. The expression level of BMP4 was verified in the GSE83569 dataset.

### Function enrichment analysis of hub DEGs

To elucidate the potential biological function of shared DEGs, Gene Ontology (GO) categories and Kyoto Encyclopedia of Genes and Genomes (KEGG) pathways analysis were performed using enrich-GO and enrich-KEGG functions in R package clusterProfiler with *p* < 0.05 as a cutoff value (Yu et al., 2012). The gene set enrichment analysis was conducted to probe the potential pathway correlated with BMP4 expression in the gene set and expression matrix of DEGs with GSVA package (Hänzelmann et al., 2013). The gene set (c2. cp.kegg.v7.4. symbols.gmt) was extracted from the Molecular Signatures Database (MsigDB) (Liberzon et al., 2015).

### Analysis of immune infiltrating cells

The ssGSEA algorithm was performed to estimate the relative composition of different immune infiltrating cells and functions on the basis of mRNA expression data in immune gene sets. The Wilcoxon test was performed to evaluate the fraction of immune infiltrating cells and the results were shown by boxplots. Spearman correlation analysis was utilized to evaluate the relationship with BMP4.

### Correlation analysis between BMP4 and m6A regulators

To further investigate the m6A regulation mechanism of OSL, Pearson correlation analysis was utilized to assess the relationship between BMP4 and 22 m6A-related regulators referred from literature (Li et al., 2019) including seven m6A writers (METTL14, WTAP, METTL3, RBM15, LRPPRC, ZC3H13, and CBLL1), two m6A erasers (ALKBH5, FTO), and 13 m6A readers (YTHDC1, YTHDF3, ELAVL1, HNRNPC, IGF2BP2, YTHDF2, IGF2BP3, YTHDC2, IGF2BP1, YTHDF1, HNRNPA2B1, FMR1, and RBMX).

## Results

### Identification of the potential shared DEGs

**FIGURE 1:**
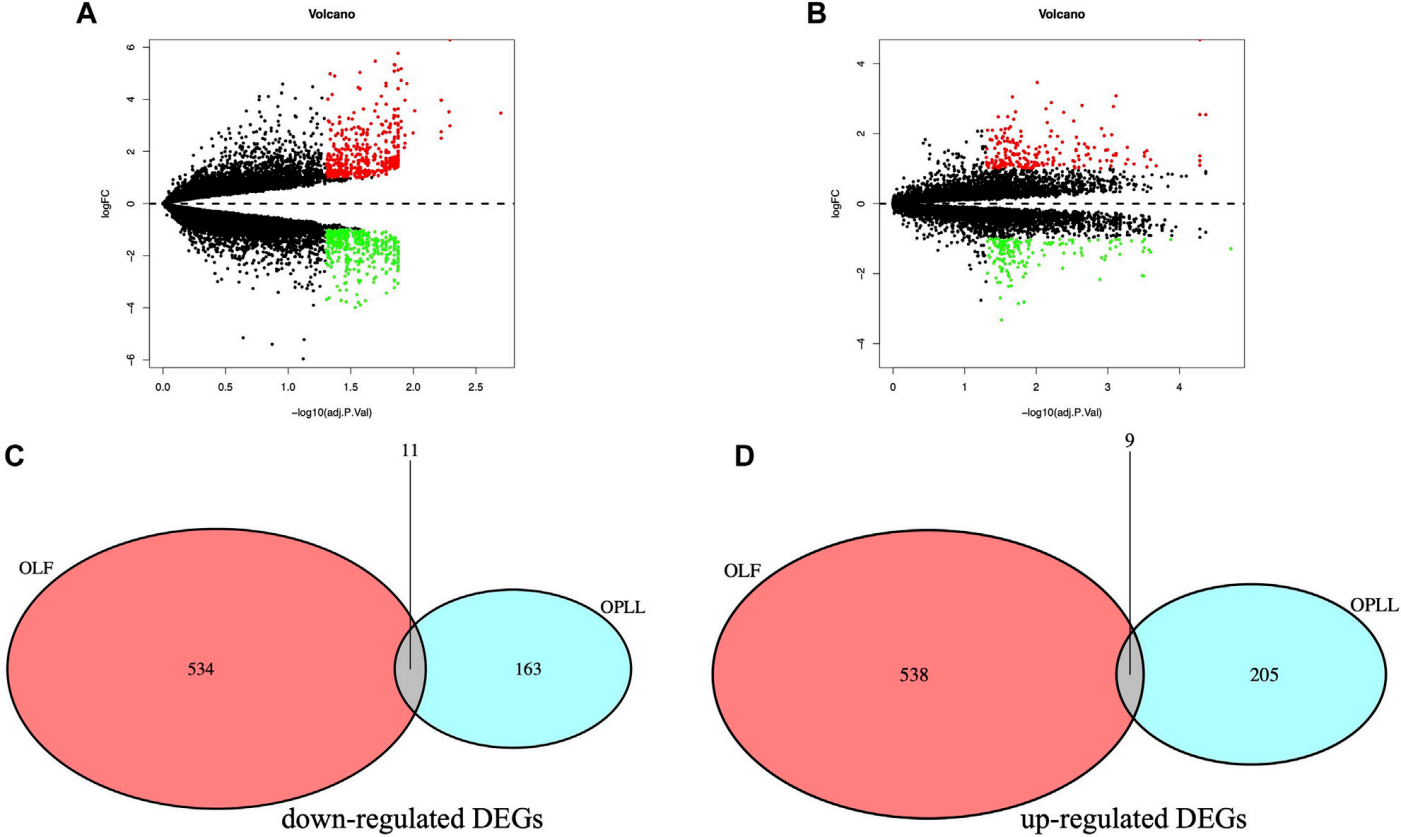
**(A)** Volcano plot of DEGs in merged OPLL datasets. **(B)** Volcano plot of DEGs in OLF dataset. **(C)** Venn plot of the down-regulated DEGs. **(D)** Venn plot of the up-regulated DEGs.

Based on the cutoff criteria, a total of 440 DEGs were screened in the combined OPLL datasets with 242 up-regulated DEGs and 198 down-regulated DEGs (Figure 1A). Besides, a total of 1,092 DEGs were screened from the GSE106253 dataset with 547 up-regulated DEGs and 545 down-regulated DEGs (Figure 1B). The shared DEGs were obtained after overlapping including nine up-regulated DEGs and 11 down-regulated DEGs (Figures 1C,D).

### PPI network construction and shared biomarker identification

**FIGURE 2:**
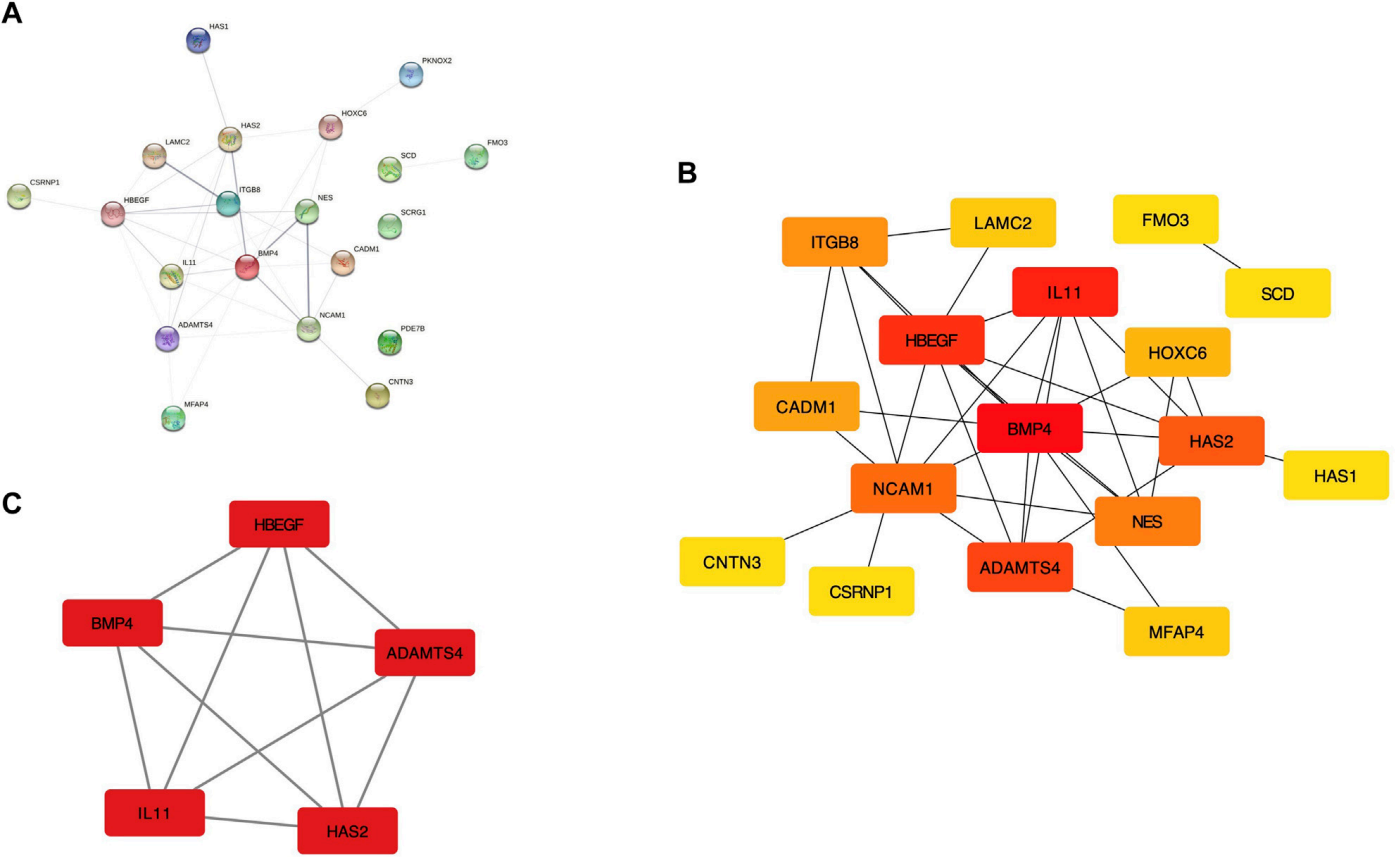
The regulation network of shared DEGs. **(A)** The PPI network with identified DEGs in STRING. **(B)** PPI network displayed in Cytoscape software. **(C)** The sub-network recognized by MCODE with the highest connectivity.

**FIGURE 3:**
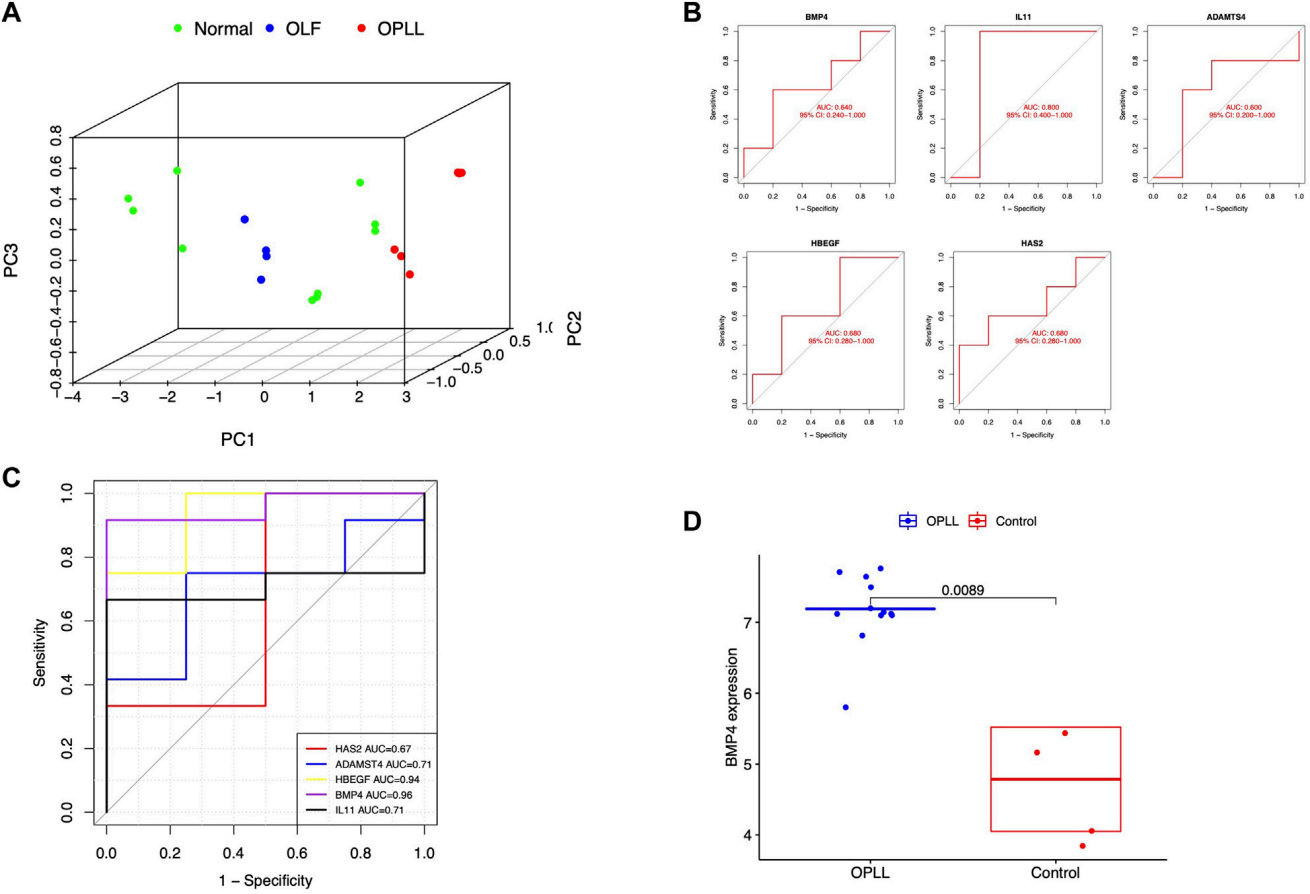
**(A)** Principal component analysis for hub genes. **(B)** Validation of hub genes in the external OLF dataset **(C)** Validation of hub genes in the external OPLL dataset **(D)** Verification of the expression level of BMP4 in the external OPLL dataset.

After eliminating the unconnected genes, the PPI network was constructed and visualized in the Cytoscape (Figures 2A,B). The critical functional module, including five hub DEGs (BMP4, ADAMTS4, HBEGF, IL11, and HAS2) was selected by MCODE. BMP4 was identified as the core gene with the highest MCC score. (Figure 2C). PCA revealed the hub genes could well distinguish the OLF and OPLL samples from normal samples (Figure 3A). To verify the diagnostic performance of the shared hub genes, the ROC analysis was performed in the external OPLL and OLF datasets. The bootstrap corrected AUC values in ROC curves indicated high accuracy of predictive value in the hub genes (Figures 3B,C). Compared with the control group, the expression level of BMP4 was higher in the OPLL group (Figure 3D), and the result confirmed that BMP4 was upregulated in the OPLL.

### Functional enrichment analysis

**FIGURE 4:**
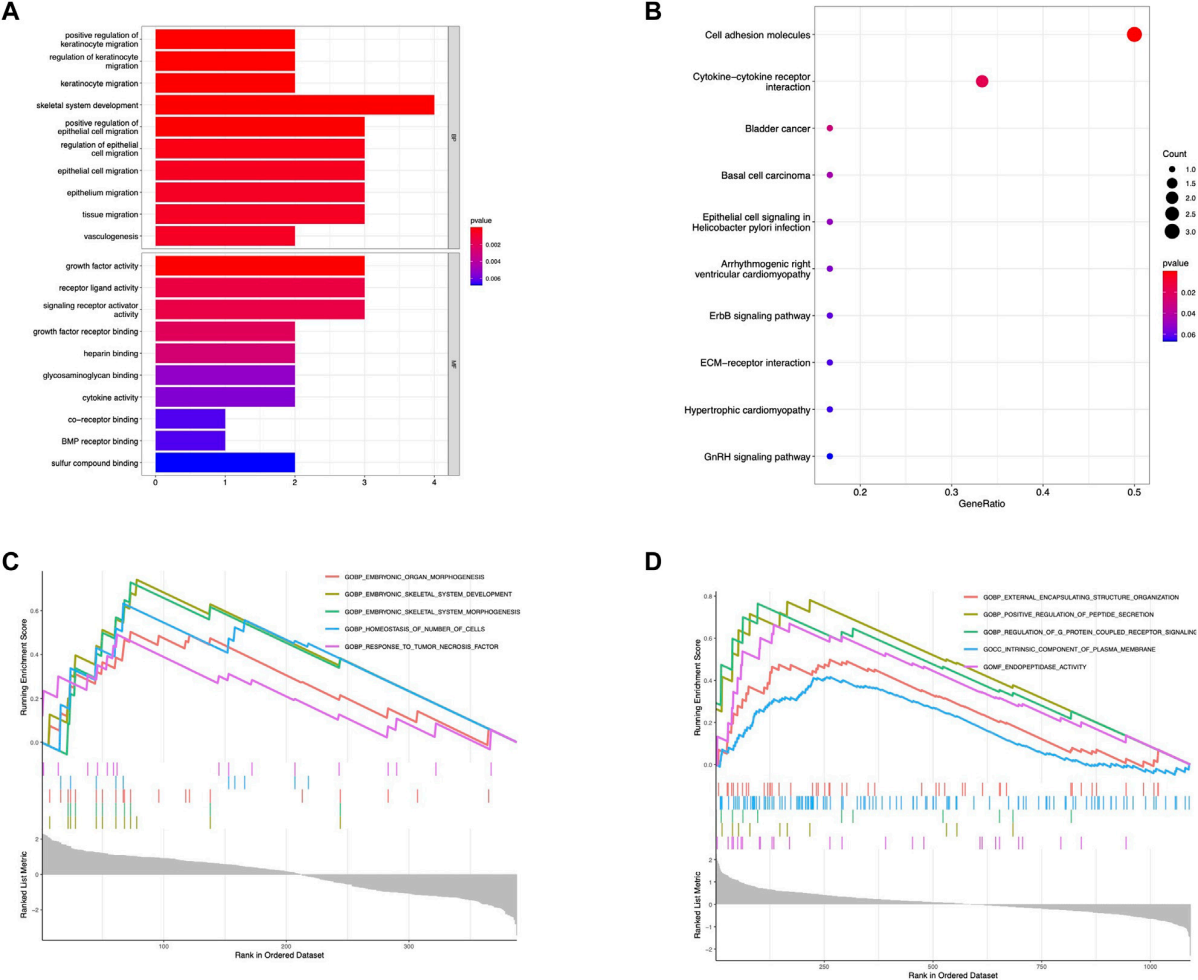
Functional enrichment analysis. **(A)** GO enrichment analysis of hub genes. **(B)** KEGG enrichment analysis of hub genes. **(C)** Single gene GSEA for BMP4 in OPLL. **(D)** Single gene GSEA for BMP4 in OLF.

Additionally, we further perform multiple enrichment analyses to explore the potential pathways of the hub gene. The GO analysis indicated that the shared hub genes were enriched in several GO terms including skeletal system development, growth factor activity, cytokine activity, and BMP receptor binding (Figure 4A). KEGG pathway analysis also demonstrated several represented signaling pathways related to the osteogenesis process, such as cytokine-cytokine interaction, JAK-STAT signaling pathway, and TGF-β signaling pathway (Figure 4B). The single-gene (BMP4) GSEA analysis in OPLL revealed significantly enriched terms were the same as GO analysis except for TNF signaling pathway while a series of pathways related to plasma membrane transport and communication were identified in OLF (Figures 4C,D).

### Correlation between BMP4 and immune cell landscape

**FIGURE 5:**
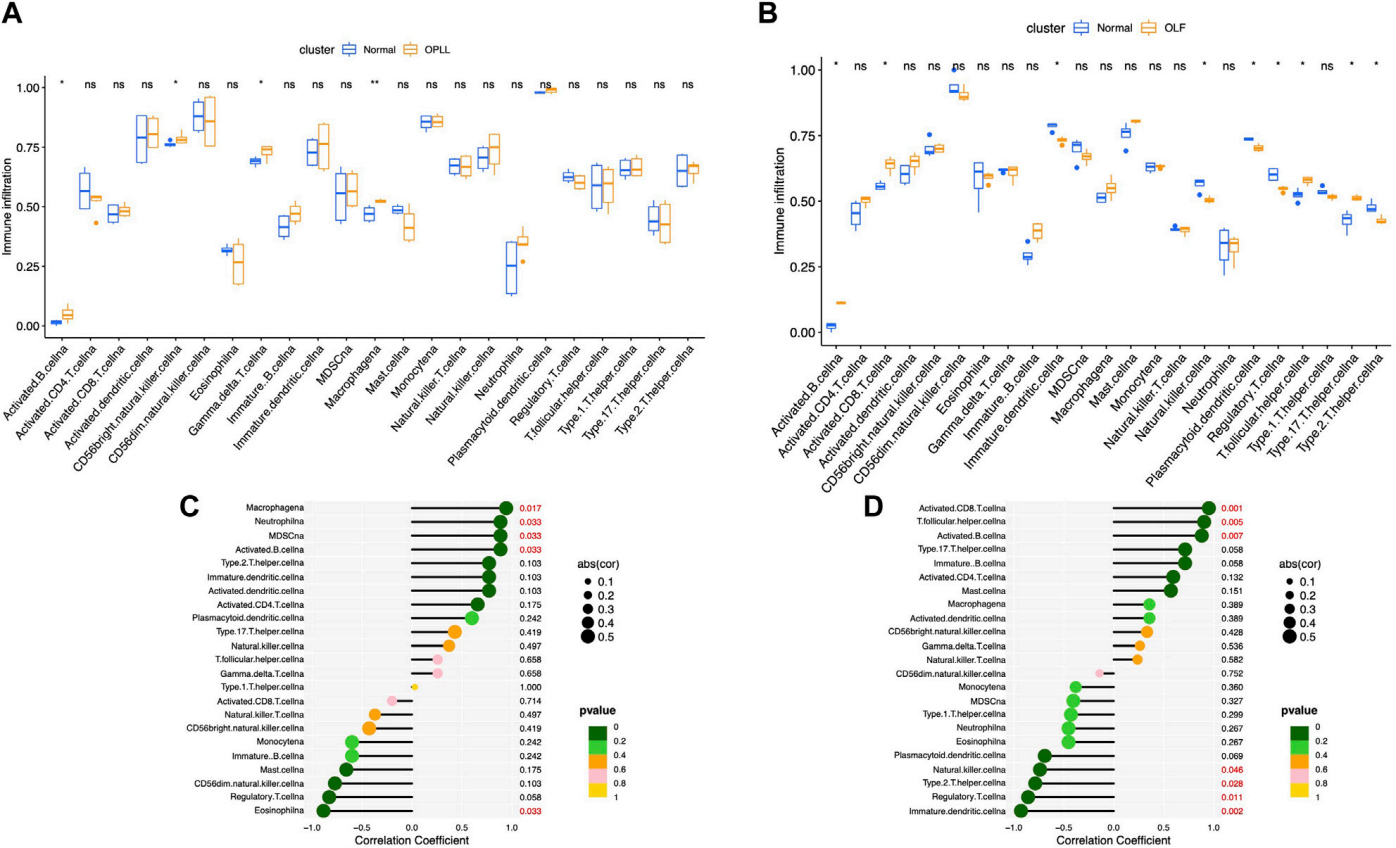
Analysis of immune infiltrating cells. **(A)** Boxplots of the fraction of immune infiltrating cells in OPLL. **(B)** Boxplots of the fraction of immune infiltrating cells in OLF. **(C)** Correlation between the immune infiltrating cells and BMP4 in OPLL. **(D)** Correlation between the immune infiltrating cells and BMP4 in OLF.

The landscapes of immune cell infiltration in OLF and OPLL were estimated by the ssGESA algorithm. The Wilcoxon test revealed four significantly altered immune subsets between OPLL and normal samples including activated B cells, NK CD56 bright cells, gamma delta T cells, and macrophages (Figure 5A). Moreover, nine significantly altered immune subtypes were detected between OLF and normal samples (Figure 5B). Activated B cells were both significantly altered in the OLF and OPLL. Additionally, we found that four immune subsets (macrophages, neutrophils, MDSCs, and activated B cells) were positively correlated with BMP4, while eosinophils were negatively correlated with BMP4 in OPLL (Figure 5C). In OLF samples, BMP4 had a positive association with activated CD8 T cells, T follicular helper cells, and activated B cells but a negative association with dendritic cells, immature dendritic cells, NK cells, and Th2 cells (Figure 5D). The results elucidated that BMP4 might participate in the B cell immune response in OSL.

### Correlation between BMP4 and m6A methylation factors

**FIGURE 6:**
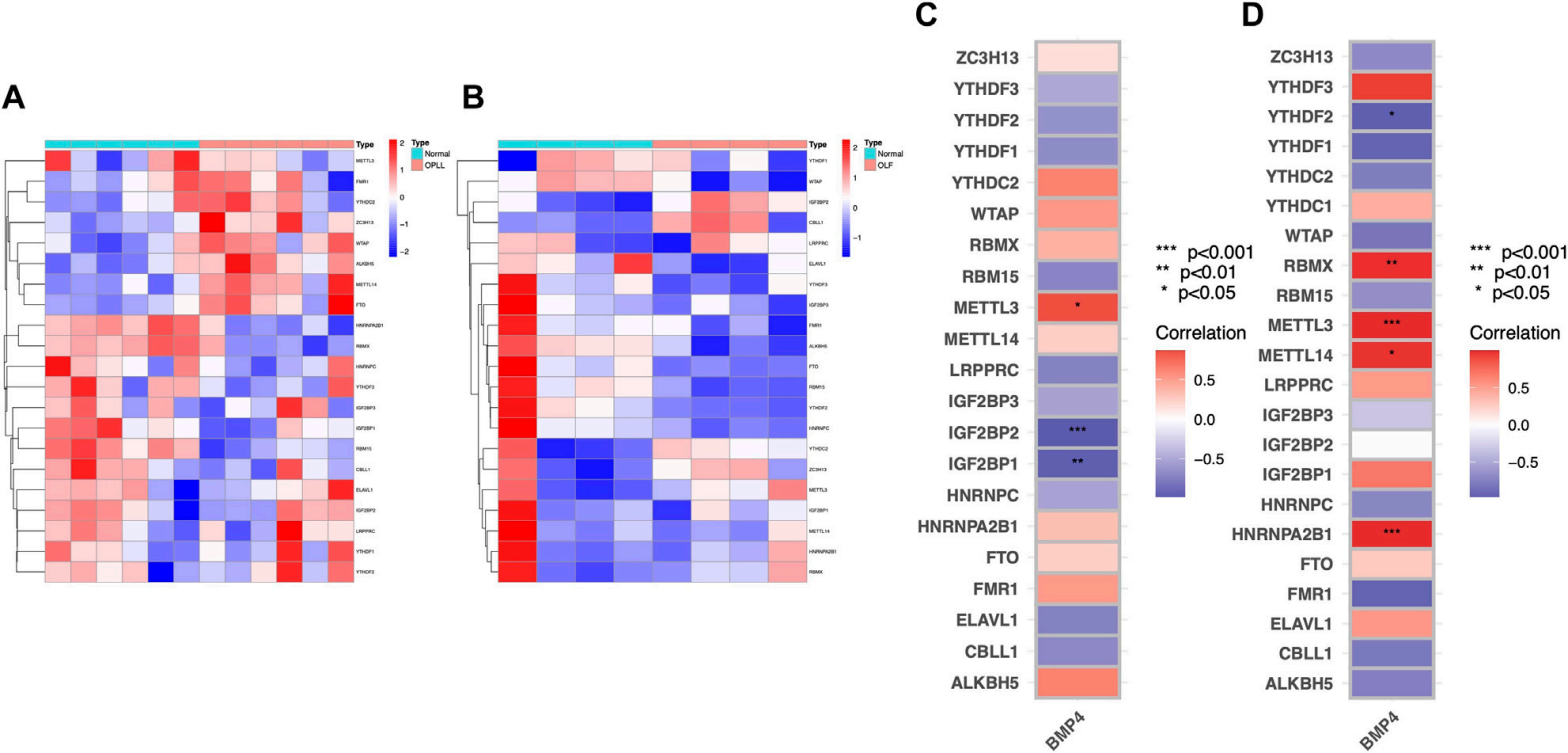
Correlation analysis of BMP4 and m6A modification factors. **(A)** The heatmap of m6A modification factors in OPLL. **(B)** The heatmap of m6A modification factors in OLF. **(C)** Correlation between m6A modification factors and BMP4 in OPLL. **(D)** Correlation between m6A modification factors and BMP4 in OLF.

The expression pattern of m6A regulation factors was shown in Figures 6A,B. Based on the OPLL datasets, we detected that three m6A methylation factors (METTL3, IGF2BP2, and IGF2BP1) were significantly correlated with BMP4. BMP4 was positively correlated with METTL3 and negatively correlated with IGF2BP2 and IGF2BP1 in OPLL (Figure 6C). Furthermore, we also found that BMP4 was significantly associated with HNRNPA2B1, METTL14, METTL3, and RBMX in OLF (Figure 6D). METTL3, as a writer in RNA methylation modification, might regulate the expression of BMP4 in the progression of OSL.

## Discussion

OPLL and OLF are both considered to be intractable and multifactorial diseases, which imposed a heavy burden on society. Numerous candidate genetic loci have been identified in recent years. However, few studies focus on the shared genetic mechanism and biomarkers. In the present study, five hub DEGs were screened from the PPI network and BMP4 was identified as the core gene in the key ossification regulating network. BMP4, a well-known osteogenic factor, has been identified to be correlated with OPLL susceptibility in the Asian population (Furushima et al., 2002; Ren et al., 2012). Nevertheless, little is known about the BMP4-related pathogenesis in OSL.

The enriched GO term was mainly implicated in growth factor activity, cytokine activity, and skeletal system development. KEGG analysis suggested that the shared hub DEGs were primarily enriched in specific pathways including the TGF-β signaling pathway, cytokine-cytokine interaction, and JAK-STAT signaling pathway. Up to date, immune and growth factor-related cytokines have been confirmed to be the essential molecules in the progression of OLF and OPLL (Ren et al., 2013; Yan et al., 2017; Qu et al., 2022; Yayama et al., 2022). The abnormal activation of TGF-β/BMPs signaling has been universally acknowledged as a classic pathway in the etiology of OSL (Qu et al., 2017; Yan et al., 2017). Moreover, the JAK-STAT pathway has been verified to accelerate the osteogenic differentiation of the spinal ligament cells (Fan et al., 2007; Chen et al., 2018). Single-gene GSEA elucidated potential functions of BMP4 in OLF and OPLL respectively, such as the TNF response and G protein-coupled signaling pathway, which were reported to be implicated in the ossification. LGR5, a leucine-rich G-protein coupled receptor, has a positive effect on the osteogenesis process of OLF (Yang et al., 2022). On one hand, numerous studies have proven that tumor necrosis factor receptor-associated factor 6 (TRAF6) might be a plausible therapeutic target that suppresses osteoblastic differentiation in the BMP2-mediated ectopic ossification of OPLL (Tsukahara et al., 2006). On the other hand, previous research has illustrated the TNF-dependent cytokine cascade in OLF (Ren et al., 2013).

Local inflammation is thought to be a vital origin in the pathogenesis of OSL, similar to the roles that play in other phenotypes of ectopic ossification and the bone remodeling process. In this research, we identified differentially expressed immunocytes in both initial innate immunity and subsequent adaptive immunity to reveal the pathogenesis of OSL. Our results showed a higher proportion of activated B cells in both OPLL and OLF samples, which is also positively associated with the expression of BMP4. Admittedly, B cells inhibit the osteogenesis in rheumatoid arthritis by suppressing the osteoblast differentiation (Sun et al., 2018). Activation of the BMP2 signal was induced by the macrophage-related cytokines in spinal ligament cells (Tsukahara et al., 2006; Ren et al., 2013). However, the interaction between adaptive immunity and BMP4 needs to be further explored.

As the most abundant form of RNA epigenetic modification in eukaryotic cells, m6A was reported to play an important role in RNA metabolism. Accumulating evidence has confirmed the novel function of m6A regulators in the mechanism of bone homeostasis (Huang et al., 2021; Xie et al., 2021). Recently, increasing studies revealed the relationship between m6A disorder and the ectopic ossification process in spinal ligament tissue. METTL3, the m6A methyltransferase, was found to promote the osteogenic process in the posterior ligament tissue *via* the USP8 regulation axial (Yuan et al., 2021). Besides, another group indicated that overexpression of m6A demethylase ALKBH5 resulted in enhancing the osteogenic effect of ligamentum flavum cells *via* demethylation of BMP2 (Wang et al., 2020). In this study, we found the correlation between BMP4 and METTL3 was positive not only in OLF but also in OPLL samples. Thus, we deduced that METTL3 is a momentous methyltransferase in the development of OSL, involved in the BMP4-directed osteogenesis process.

However, some limitations inevitably exist in this research. First, although we included all available datasets in the GEO database, the amount of data is still limited. More multicenter external datasets and further experimental research are required to explore the specific molecular mechanisms of the hub genes. Second, the association between clinical parameters and hub genes should be evaluated in further research. Considering the limitation of this study, the present study hopes to preliminary provide a meaningful perspective on the shared biomarkers related to ectopic ossification in the spinal ligament.

## Conclusion

In conclusion, the present research is the first to identify the distinct shared biomarkers and pathways of OLF and OPLL at the transcriptional level. Integrating multiple datasets and bioinformatic methods, we detected that BMP4 was a core biomarker in the shared mechanism of OPLL and OLF and correlated with B cell response and methylation modification of METTL3. Our findings provide novel insights into the shared pathogenesis of OPLL and OLF to unveil potential therapeutic targets.

## Data Availability

Publicly available datasets were analyzed in this study. This data can be found here: The datasets were downloaded from the Gene Expression Omnibus (GEO) database (http://www.ncbi.nlm.nih.gov/gds/).
